# Post Tonsillectomy Pain: Can Honey Reduce the Analgesic Requirements?

**DOI:** 10.5812/aapm.9246

**Published:** 2013-07-01

**Authors:** Peyman Boroumand, Mohammad Mahdi Zamani, Masoumeh Saeedi, Omid Rouhbakhshfar, Seyed Reza Hosseini Motlagh, Fatemeh Aarabi Moghaddam

**Affiliations:** 1 Department of Otolaryngology, Zahedan University of Medical Science, Zahedan, Iran; 2 Department of Anesthesiology, Firoozgar Hospital, Iran University of Medical Sciences (IUMS), Tehran, Iran; 3 Students’ Scientific Research Center, School of Medicine, Tehran University of Medical Sciences, Tehran, Iran

**Keywords:** Analgesics, Honey, Pain, Postoperative, Tonsillectomy

## Abstract

**Background:**

Tonsillectomy with or without adenoidectomy is one of the most common surgical procedures performed worldwide, especially for children. Oral honey administration following tonsillectomy in pediatric cases may reduce the need for analgesics via relieving postoperative pain.

**Objectives:**

The aim of this study was to evaluate the effects of honey on the incidence and severity of postoperative pain in patients undergoing tonsillectomy.

**Patients and Methods:**

A randomized, double blind, placebo controlled study was performed. One hundred and four patients, who were older than eight, and were scheduled for tonsillectomy, were divided into two equal groups, honey and placebo. Standardized general anesthesia, and postoperative usual analgesic, and antibiotic regimen were administrated for all patients. Acetaminophen plus honey for the honey group, and acetaminophen plus placebo for the placebo group were given daily. They began to receive honey or placebo when the patients established oral intake.

**Results:**

The difference between acetaminophen and acetaminophen plus honey groups was statistically significant both for visual analogue scale (VAS), and number of painkillers taken within the first three postoperative days. The consumption of painkillers differed significantly in every five postoperative days. No significant difference was found between groups regarding the number of awaking at night.

**Conclusions:**

Postoperative honey administration reduces postoperative pain and analgesic requirements in patients after tonsillectomy. As the side effects of honey appear to be negligible, consideration of its routine usage seems to be beneficial along with routine analgesics.

## 1. Background

Infectious and inflammatory diseases which involve pharynx, tonsils and adenoid, are of great importance among children’s diseases. They mostly result in two common pediatric surgeries: tonsillectomy, and adenotonsillectomy (1). Modern anesthesiology has been focused on perioperative period along with intraoperative period (2). Post tonsillectomy pain is a common problem of this surgery (3). Severe pain can cause reduction in oral intake, dehydration, impairment or latency in recovery after surgery. The most common administered drug for reducing post tonsillectomy pain is acetaminophen but it cannot relief pain completely (4). In a questionnaire study including 52 children following tonsillectomy, 90% of the children receiving paracetamol as pain medication, experienced pain at home during the first 24 h after the operation, and in many cases the pain still remained seven days after the operation (5). Thus along with acetaminophen, other drugs are used for lowering post tonsillectomy pain, such as nonsteroidal anti-inflammatory drugs (NSAIDS), opiates, injectable steroids, topical anesthetizing sprays, fibrin glue, fusa fungine or sucralfate (4, 6, 7), but the efficacy and side effects of these agents necessitate more surveys to find post tonsillectomy pain relieving drugs besides acetaminophen. It is a long time that honey is used for its biological and therapeutic effects. About 400 years before Jesus Christ, Hypocrite used honey for wound healing, especially the ones on foot. Even ancient Egyptians used honey for treatment of the corneal and conjunctival inflammation, and burns at about 5000 years ago (8, 9). In modern medicine, honey has been used successfully in treatment of burns, split-thickness skin graft donor site, necrotizing fasciitis, operation site infection in neonates, skin injuries, pressure induced wounds, infective wounds, infected surgical wounds, diabetic wounds, malignancy related wounds, gangrene, osteomyelitis, gingivitis, periodontal diseases, bullous keratopathy, and corneal lesions (8, 10-13). Honey speeds up healing in chronic wound by stimulating production of inflammatory cytokines (CK) from monocytes (14) and keratinocytes (15). It is shown that honey motivates monocytes to secret CKs like Interleukin (IL)-1B, IL-6, and tumor necrosis factor (TNF)-alpha. These mediators play an important role in healing, and tissue repair (16, 17). Administration of oral honey after tonsillectomy in children decreases the need to analgesics via pain reduction after surgery (9). In previous studies, there is no report for honey’s side effects in wound healing (18). Human allergy to honey is rare, but an allergic reaction to proteins and allergens of honey is possible (10). Honey rarely contains clostridial spores which cause wound botulism, however there is no report through many researches when open wound is being sterilized before honey usage (19, 20).

## 2. Objectives

The aim of this study is to investigate the effect of honey administration on pain along with acetaminophen, following pediatric tonsillectomy or adenotonsilectomy.

## 3. Patients and Methods

The study design was randomized, double blind placebo-controlled clinical trial. One hundred and two patients, aging from 8 to 15 years, who had been referred to otolaryngeal clinic of Khatam-ol-anbia hospital (a referral and educational hospital) in Zahedan, Iran were recruited. This study was approved by the regional ethics committee of Hospital. Inform consent was obtained from each parent. All subjects who had the indication of tonsillectomy, and had undergone classic tonsillectomy with or without adenoidectomy were included in this study. For all the subjects, endotracheal intubation and anesthesia method was the same (Fentanyl 2 µg/kg, Lidocaine 1 mg/kg, Thiopental Na 5 mg/kg, and Atracurium 0.6 mg/kg, for induction, and a mix of nitrous oxide (N_2_O)/oxygen (O_2_) 50%/50%, Sevoflurane 3.3%, and Atracurium 0.2 mg/kg every 30 minutes were used as maintenance). Subjects who had allergy to honey or acetaminophen, disliked to consume honey, were affected to diabetes mellitus, had abnormal coagulopathy or any extra surgery were excluded from the study. The acetaminophen group was treated with antibiotic (amoxicillin 40 mg/kg), acetaminophen (15 mg/kg/dose maximum 5 times a day), and as placebo, a tea spoon (5 ml) of sugar syrup in honey-like concentration, consistency and coloring (no artificial color or flavor was added). Acetaminophen-plus-honey group was treated with antibiotic (amoxicillin 40 mg/kg), acetaminophen (15 mg/kg/dose maximum 5 times a day), and a tea spoon (5 ml) of honey when they woke up. Parents were asked to give acetaminophen to their children. Acetaminophen was used as tablet 325 mg after 24 hour post tonsillectomy according to patient’s request and severity of pain. Administration of placebo and honey was started when the patient was able to have oral intake and continued for 5 days. Six hours after the operation the patients began to have oral intake, and all of them tolerated PO. To prevent bias, the study was designed double blinded, and none of the patients and their parents knew what their group is, as well as the surgeon. From the first to 5th day after the operation, visual analogue scale (VAS) was applied for subjective assessment of postoperative pains by the parents every day, and also 4 hours after acetaminophen consumption, while the numbers of painkillers taken daily and awaking at night due to pain were used for objective assessment. The Statistical Package of Social Science version 15.0 (SPSS, Chicago, Illinois, USA) was used for data analysis. Statistical significance was noted for p value of ≤ 0.05. Chi-square test was used to compare frequencies and distributions, and t-test was used to compare quantitative data and means between groups. Data were expressed as mean ± SD.

## 4. Results

The study consisted of 52 subjects in case group and 52 ones in control group. Totally, 48 subjects were male (46.1%), and 56 cases were female (53.8%). There was no significant difference between groups in gender. Age range of subjects was 8 to 15 years. The average age in the case group was nine years, and in the control group was 10 years. There was no significant difference in age, between groups. From the first day to the third day after the operation, the mean pain score in case group (honey) was significantly less than control group (placebo) (Table 1). In the 4th day after the operation, pain score in case group was 2.5 ± 0.28 and in control group was 2.6 ± 0.3, which was not statistically significant. Similarly, in the fifth day postoperative, there was no significant difference between groups in pain scores. From the first to fifth day after the operation, the need to analgesics was significantly lower in honey group compared to the placebo (Table 2). There was not any significant difference between groups in awaking at night because of pain. There was not any allergic reaction to honey in this study.

**Table 1.** Pain Scores of Groups in the 1st, 2nd, and 3rd DayAfter Tonsillectomy (Variables Are Expressed as Mean ± SD)

Group	First day		Second day		Third day	
	Pain Score	P value	Pain Score	P value	Pain Score	P value
Placebo	5.4 ± 0.56	< 0.001 ^a^	4.6 ± 0.49	< 0.001 ^a^	3.8 ± 0.47	< 0.01 ^a^
Honey	3.8 ± 0.69		3.2 ± 0.43		2.6 ± 0.42	

^a^P < 0.05

**Table 2.** Number of Painkillers Taken After Tonsillectomy (Variables Are Expressed as Mean ± SD)

	First day		Second day		Third day		Forth day		Fifth day	
	Painkiller	P value	Painkiller	P value	Painkiller	P value	Painkiller	P value	Painkiller	P value
Placebo	3.52 ± 1.6	< 0.01 ^a^	3 ± 1.12	< 0.01 ^a^	2.58 ± 1.09	< 0.01 ^a^	1.81 ± 1.14	0.04 ^a^	1.15 ± 0.99	< 0.01 ^a^
Honey	1.81 ± 1.16		1.5 ± 1.05		1.15 ± 0.95		0.61 ± 0.77		0.21 ± 0.45	

^a^P < 0.05

## 5. Discussion

Medical practitioners have become increasingly concerned about adequate pain management because of the increasing number of complex outpatient procedures, and ambulatory surgeries (21). Nowadays honey is one of the remedies being used widespread, and is not palatable even for children. In a meta-analysis held by Wijesinghe et al. in 2009, it was reported that those studies indicated markedly greater efficacy of honey compared with alternative dressing treatments for superficial or partial thickness burns (22). Macroscopic and microscopic studies under in vivo assessment suggested that the topical application of honey influences the various phases of burn and wound healing by anti-inflammatory agents, and growth factors from monocytes, and the mechanisms are unclear yet (14). The data show that the wound healing properties of honey include stimulation of tissue growth, enhanced epithelialization, and minimized scar formation. These effects are ascribed to honey’s acidity, hydrogen peroxide content, osmotic effect, nutritional and antioxidant contents, stimulation of immunity, and to unidentified compounds. Prostaglandins and nitric oxide play a major role in inflammation, microbial killing, and the healing process. Honey was found to lower prostaglandin levels and elevate nitric oxide end products. These properties might help to explain some biological and therapeutic properties of honey, particularly as an antibacterial agent or wound healing (23). The most common morbidities after tonsillectomy with or without adenoidectomy are bleeding, edema, nausea, vomiting, poor oral intake, and pain (24). Despite advances in anesthetic and surgical techniques, post tonsillectomy morbidity remains a major clinical problem. On the other hand, many studies are being performed to find treatments with fewer side effects, especially for pediatric patients who are more sensitive. Many researches have been performed to investigate different analgesics’ effects on post tonsillectomy pain, especially together with acetaminophen. In many studies, relief of early postoperative pain, in first hours of operation, was investigated (25, 26). On the other hand, many studies look into postoperative pain after recovery room; in a study held in Finland, it was shown that ketoprofen combined with paracetamol - codeine seems to provide sufficient analgesia during 10 days after surgery (27). A systematic review published in the same country revealed that no analgesic in single prophylactic dosage is enough to provide analgesia during the day of operation, thus, repeated administration, and also combination with NSAIDS, and titrated opioids are needed to reach optimal result, and guarantee freedom from pain (28). It also recommended the use of oral acetaminophen rather than rectal form, as used in our study. In a study from Turkey in 2006, the post tonsillectomy effect of honey in pain killing was surveyed for 14 days, and it was reported that pain scores in first two days after the operation were significantly less in honey group (9), compared to our study which shows this difference from the first to the third day after tonsillectomy. They also reported the reduction in taking analgesics from the first to 8th day post tonsillectomy. Similarly, our study shows significant difference of using analgesics in all five days of study and by using honey, the need for using analgesics decreased. Oral administration of honey after wake up, following tonsillectomy or adenotonsilectomy can reduce postoperative pain in pediatric patients, and may substantially decrease the need for analgesics during taking honey in this challenging group. More studies are necessary to be performed to investigate microscopic mechanisms of honey pain relieving effects. There were some limitations in this study such as: disagreement of parents in continuing their cooperation, the child’s dislike for eating the placebo or honey, misunderstanding of the details of VAS by parents.
